# Effects of Direct Renin Inhibition on Myocardial Fibrosis and Cardiac Fibroblast Function

**DOI:** 10.1371/journal.pone.0081612

**Published:** 2013-12-11

**Authors:** Hui Zhi, Ivan Luptak, Gaurav Alreja, Jianru Shi, Jian Guan, Nicole Metes-Kosik, Jacob Joseph

**Affiliations:** 1 VA Boston Healthcare System, West Roxbury, Massachusetts, United States of America; 2 Boston University School of Medicine, Boston, Massachusetts, United States of America; 3 Brigham and Womens Hospital, Harvard Medical School, Boston, Massachusetts, United States of America; Albert Einstein College of Medicine, United States of America

## Abstract

Myocardial fibrosis, a major pathophysiologic substrate of heart failure with preserved ejection fraction (HFPEF), is modulated by multiple pathways including the renin-angiotensin system. Direct renin inhibition is a promising anti-fibrotic therapy since it attenuates the pro-fibrotic effects of renin in addition to that of other effectors of the renin-angiotensin cascade. Here we show that the oral renin inhibitor aliskiren has direct effects on collagen metabolism in cardiac fibroblasts and prevented myocardial collagen deposition in a non-hypertrophic mouse model of myocardial fibrosis. Adult mice were fed hyperhomocysteinemia-inducing diet to induce myocardial fibrosis and treated concomitantly with either vehicle or aliskiren for 12 weeks. Blood pressure and plasma angiotensin II levels were normal in control and hyperhomocysteinemic mice and reduced to levels lower than observed in the control group in the groups treated with aliskiren. Homocysteine-induced myocardial matrix gene expression and fibrosis were also prevented by aliskiren. *In vitro* studies using adult rat cardiac fibroblasts also showed that aliskiren attenuated the pro-fibrotic pattern of matrix gene and protein expression induced by D,L, homocysteine. Both *in vivo* and *in vitro* studies demonstrated that the Akt pathway was activated by homocysteine, and that treatment with aliskiren attenuated Akt activation. In conclusion, aliskiren as mono-therapy has potent and direct effects on myocardial matrix turnover and beneficial effects on diastolic function.

## Introduction

Heart failure with preserved ejection fraction (HFPEF) is increasing in prevalence and is associated with significant morbidity and mortality [1]. Myocardial fibrosis resulting in increased myocardial stiffness and diastolic dysfunction is a major pathophysiological component of HFPEF [2]. Activation of the renin-angiotensin-aldosterone system (RAAS) has been shown to promote myocardial fibrosis and diastolic dysfunction [3]. However, recent clinical trials of the use of angiotensin converting enzyme inhibitors (ACEI) and angiotensin II type 1 receptor blockers (ARBs) have not shown a benefit in HFPEF patients [4]–[6]. One of the potential reasons for the lack of efficacy of ACEI and ARB in clinical trials is that the anti-fibrotic effects of these agents alone or in combination require supra-therapeutic dose ranges [7]. Aliskiren, as a direct renin inhibitor, functions through inhibition of angiotensin II effects as well as angiotensin II-independent effects mediated via the (pro)renin receptor as shown in recent studies [8], [9]. To examine whether aliskiren has potent and direct anti-fibrotic effects, we studied the effects of aliskiren in a unique model of hyperhomocysteinemia-induced myocardial fibrosis and diastolic dysfunction, which is not associated with hypertension or left ventricular hypertrophy. We observed that aliskiren as mono-therapy has potent and direct effects on myocardial matrix turnover and thereby on diastolic function, suggesting that direct renin inhibition might be an effective therapy in HFPEF.

## Materials and Methods

### Animal model

The study was conducted in strict accordance with the recommendations in the Guide for the Care and Use of Laboratory Animals of the National Institutes of Health. All procedures in this study were approved by the Institutional Animal Care and Use Committee of Boston University School of Medicine (Protocol # AN-15057) and Harvard Medical School (Protocol # 04783). Male C57BL6 mice (8–10 weeks old) were purchased from Charles River Laboratories (Boston, MA, USA) and were maintained in our institutional Division of Laboratory Animal Medicine on a 12∶12 light-to-dark cycle with free access to chow and water. Osmotic mini-pumps for delivery of aliskiren (drug provided by Novartis Pharmaceuticals) were implanted subcutaneously in the dorsal chest under anesthesia. Either vehicle, or aliskiren in the doses of 0.5 mg/kg/day, 5 mg/kg/day, or 50 mg/kg/day, was delivered via osmotic mini-pump. Since aliskiren is designed to specifically inhibit human renin, higher doses are required to inhibit murine renin, and 50 mg/kg/day is a widely accepted dose to provide adequate inhibition of murine rennin [10]. Immediately after implantation of mini-pumps, animals were started on control amino-acid defined diet or hyperhomocysteinemia (Hhe)-inducing diets used in our prior studies [11]. Diets were continued for a total of 12 weeks. Since osmotic mini-pumps can deliver drug only for 6 weeks, repeat surgery and implantation of new osmotic mini-pumps to deliver drug was done at 6 weeks. Blood pressure was measured at 12 weeks of treatment in awake animals using a noninvasive computerized tail-cuff system (BP-2000 Visitech Systems, Apex, NC). Euthanasia was performed by inducing general anesthesia using ketamine and xylazine after 12 weeks of treatment and blood was collected for measurement of angiotensin II and hearts were collected for functional, histologic and biochemical analysis.

### Histological analysis of myocardial remodeling

Coronal sections of ventricular myocardium were fixed in 10% neutral buffered formalin, and serial sections (5 μm) were stained with hematoxylin and eosin for estimating myocyte size, and with Picrosirius red for estimating fibrillar collagen. Perivascular collagen, coronary arteriolar wall thickening, interstitial collagen volume fraction, and myocyte size were measured as described previously [12].

### Measurement of cardiac function

Cardiac function was measured *ex vivo* utilizing isolated perfused Langendorff heart preparations [12]. Age matched mice were used as a control group. Briefly, mice were heparinized (100 U, intraperitoneally) and anesthetized by sodium pentobarbital (150 mg/kg, intraperitoneally). The heart was excised and perfused at a constant pressure of 80 mm Hg at 37°C. The perfusate was equilibrated with 95% O2 and 5% CO2 (pH 7.4) and contained the following (in mmol/L): NaCl (118), NaHCO3 (25), KCl (5.3), CaCl2 (1.8), MgSO4 (1.2), glucose (10) and pyruvate (0.5). Hearts were paced at 7.5 Hz throughout the protocol. A water-filled balloon was inserted into the left ventricle (LV) to record ventricular pressure. After 20 min stabilization, a LV pressure–volume (P–V) relationship was obtained by stepwise increases of the balloon volumes, until the maximum LV developed pressure was reached for each heart. To correct for variation in heart size, LV volume was normalized to heart weight.

### Real-time PCR analysis

Total RNA was extracted from frozen mouse heart sections using RNeasy Fibrous Tissue Mini-columns (catalog # 74704, Qiagen Inc. Germantown, MD). High Capacity cDNA Reverse Transcription Kit (catalog # 4374966, Applied Biosystems, Foster City, CA) was utilized to make cDNA from 1 µg of RNA. Real-time PCR was performed using the TaqMan universal PCR mastermix (catalog # 4304437, Applied Biosystems, Foster City, CA) and the TaqMan inventoried FAM labeled Gene Expression assays for alpha 1 chain of type I collagen (COL1A1; catalog # Mm00801666_g1 for mouse, catalog # Rn01463848_m1 for rat), alpha 2 chain of type I collagen (COL1A2; catalog # Mm01165187_m1 for mouse, catalog # Rn00670305_m1 for rat), and alpha 1 chain of type III collagen (COL3A1; catalog # Mm01254476_m1). Data were normalized by multiplexing with the Taqman inventory VIC labeled gene expression assay for GAPDH (catalog # 4352339e for mouse, catalog # 4352338e for rat) endogenous control assay and expression was compared relative to control treated samples using the formula, 2−^ΔΔ^CT, on the Step One Applied Real-Time PCR System Instrument (Applied Biosystems). The PCR cycling conditions were 50°C for 10 minutes, 95°C for 5 minutes, followed by 40 cycles of 95°C for 15 seconds and 60°C for 1 minute. Data were analyzed using the comparative cycle threshold (Ct) method.

### Measurement of plasma angiotensin II level

Plasma angiotensin II level was measured in the Harvard Catalyst Central Laboratory utilizing a double-antibody radioimmunoassay using modification of the method described by Emmanuel et al [13]. Briefly reversed phase-extraction plasma samples and standards were incubated for 16 hours with the anti-angiotensin II antibody; ^125^I-angiotensin II was then added to compete with angiotensin II present in samples and standards for the same binding sites on the anti-angiotensin II antibody. After further incubation for 6 hours, the solid phase second antibody was added to the mixture, and the antibody-bound fraction was precipitated and counted.

### Cell Culture

Adult rat cardiac fibroblasts were isolated and cultured as described previously in publications from our laboratories [14]. Cardiac fibroblasts (passage 1) which were 80–90% confluent were serum-deprived for 24 hours before initiation of treatments. Cells were exposed to the pro-fibrotic stimulus D, L, homocysteine in the concentrations of 0 or 400 µmol/L with or without pre-exposure to aliskiren 0, 0.1, 1, or 10 µmol/L. Both cells and conditioned media were collected at the end for measurement of collagen turnover. To test the Akt signaling pathway, Wortmannin (1 µmol/L) (PI3K inhibitor, Cell Signaling Technology, Beverly, MA) and Akt Inhibitor VIII (1 µmol/L; Millipore, Billerica, MA) were utilized. Losartan (0.1 mmol/L) and Captopril (0.3 mmol/L) (Sigma-Aldrich Corp., St. Louis, MO) were used to block angiotensin II function.

### Western blot Analysis

Western blot analysis was conducted as described previously (15). Primary antibodies utilized for this study were: Collagen I (Millipore Corporation, Temecula, CA); phospho-Akt and Akt, (Cell Signaling, Beverly, MA); and GAPDH (R and D Systems, Minneapolis, MN).

### Statistical Analysis

Data were evaluated by ANOVA with a Student-Newman-Keuls post-hoc test or by t-test as appropriate using *GraphPad Prism* (Graph Pad Software, La Jolla, CA). The criterion for significance was a *p* value <0.05. Data are reported as means ± SEM.

## Results

### Effects of aliskiren on cardiac hypertrophy, blood pressure and RAS

**Figure 1:**
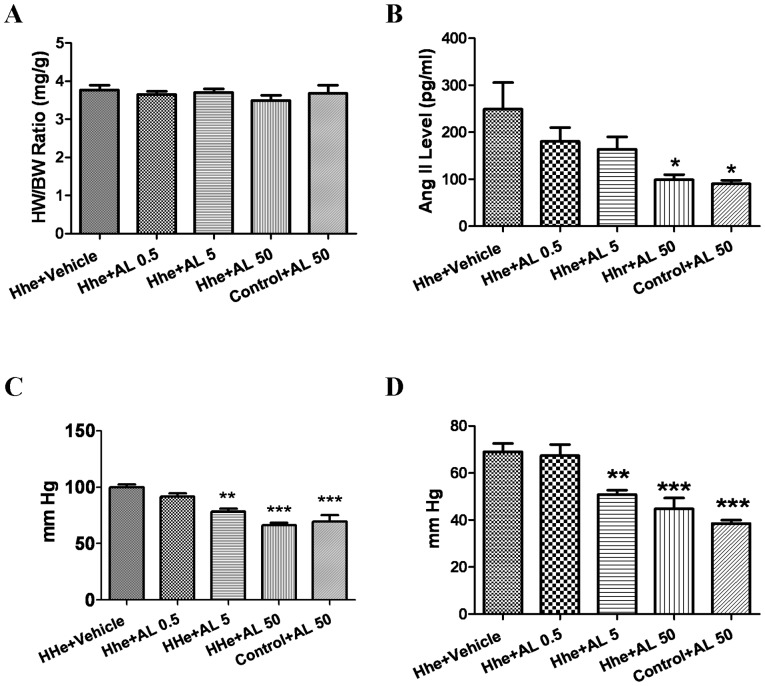
Effect of aliskiren on blood pressure and plasma angiotensin II levels. Data was obtained after 12-inducing diets and aliskiren. A. Heart weight to body weight ratios; B. Angiotensin II levels; C. Systolic blood pressure; and D. Diastolic blood pressure. HW – heart weight; BW – body weight; Hhe – Hhe-inducing diet; AL 0.5– aliskiren 0.5 mg/kg/day; AL 5– aliskiren 5 mg/kg/day; AL 50– aliskiren 50 mg/kg/day; * – p<0.05; ** – p<0.01; and ***– p<0.001.

As shown in Figure 1, the blood pressure values obtained in the Hhe group treated with vehicle were not elevated compared to those observed in normal mice [15]. Systolic and diastolic blood pressures were decreased significantly below control values in the groups given the aliskiren doses of 5 mg/kg/day and 50 mg/kg/day compared to vehicle or aliskiren 0.5 mg/kg/day. The blood pressure values in Hhe group and control group treated with aliskiren 50 mg/kg/day were similar. Heart weight to body weight ratios were similar between groups, suggesting that there was no ventricular hypertrophy in any group. Cardiomyocyte size estimated as cross-sectional diameter was similar between groups (Control: 16±0.4 µm; Hhe: 16.4±0.8 µm; Hhe + aliskiren 0.5 mg/kg/day: 16.7±0.8 µm; Hhe + aliskiren 5 mg/kg/day: 16.1±0.8 µm; and Hhe + aliskiren 50 mg/kg/day: 16.3±0.6 µm). Plasma angiotensin II levels were decreased significantly only by aliskiren 50 mg/kg/day (Figure 1).

### Aliskiren prevents Hhe-induced reactive myocardial fibrosis

**Figure 2:**
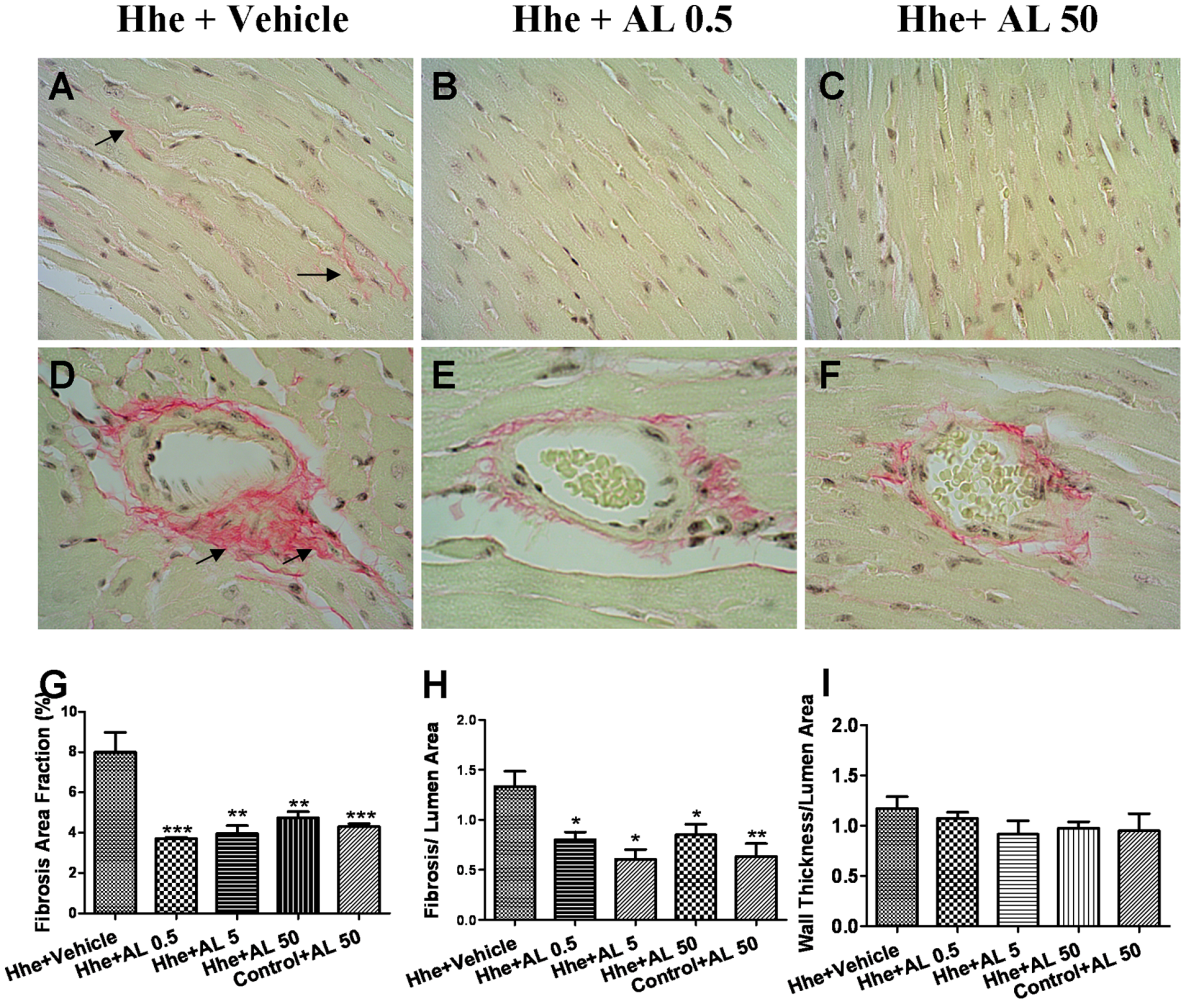
Aliskiren prevents reactive myocardial fibrosis induced by hyperhomocysteinemia. Upper panel shows representative sections from various groups demonstrating that interstitial fibrosis (panels A, B, C and G) and perivascular fibrosis (Panels D, E, F and H) are decreased by low and high dose aliskiren. Coronary arteriolar wall thickness did not vary between groups (I). Hhe – Hhe-inducing diet; AL 0.5– aliskiren 0.5 mg/kg/day; AL 5– aliskiren 5 mg/kg/day; AL 50– aliskiren 50 mg/kg/day; *p<0.05; **p<0.01; and ***p<0.001.

**Figure 3:**
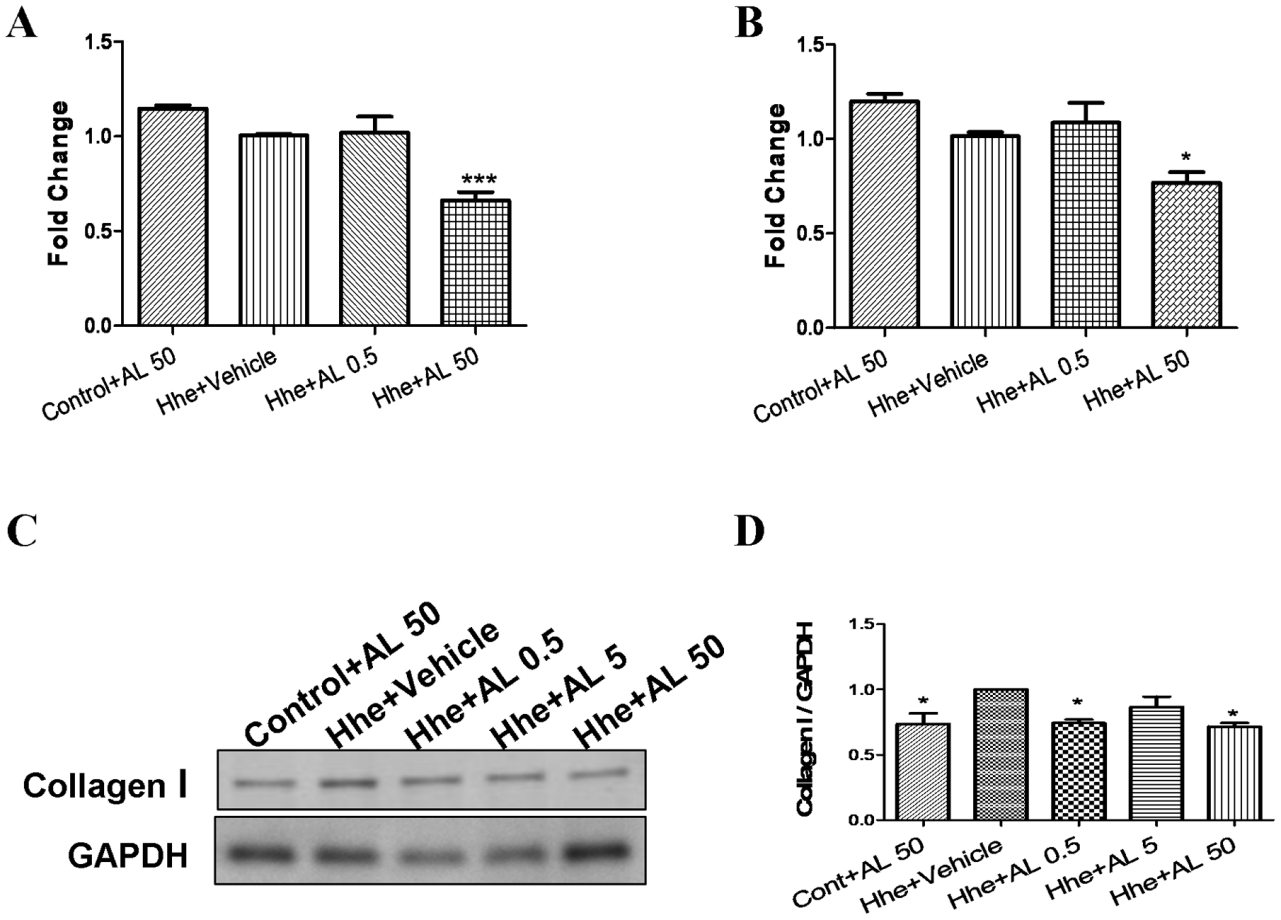
Effects of aliskiren on myocardial matrix gene expression. Real time PCR was performed with ventricular tissue obtained from mice after 12-inducing diet and different doses of aliskiren. Figure 3A demonstrates changes in expression of COL1A1(alpha 1 chain of type I collagen) and 3B shows changes in COL1A2(alpha2 chain of type I collagen) genes. Lower right panels (3C and 3D) demonstrate the results of western blotting for collagen type I in various groups. Hhe – Hhe-inducing diet; AL 0.5– aliskiren 0.5 mg/kg/day; AL 5– aliskiren 5 mg/kg/day; and AL 50– aliskiren 50 mg/kg/day. * p<0.05; and *** p<0.001.

As shown in Figure 2, Hhe-induced perivascular and interstitial fibrosis were attenuated by all three doses of aliskiren, including the lowest dose of 0.5 mg/kg/day. Coronary arteriolar wall thickness did not vary between groups (Figure 2). Figure 3 demonstrates the effects of aliskiren on expression of collagen type I, the most commonly expressed collagen in the myocardium. The expression of the genes for the alpha 1 chain (COL1A1) and alpha 2 chain (COL1A2) of type I collagen were decreased significantly by aliskiren 50 mg/kg/day compared to vehicle. The amount of collagen type I protein in the myocardium was significantly decreased by all three doses of aliskiren compared to treatment with vehicle. Hence aliskiren, even in doses that did not lower angiotensin II levels or blood pressure, prevented Hhe-induced myocardial fibrosis.

### Aliskiren prevents diastolic dysfunction induced by hyperhomocysteinemia

**Figure 4:**
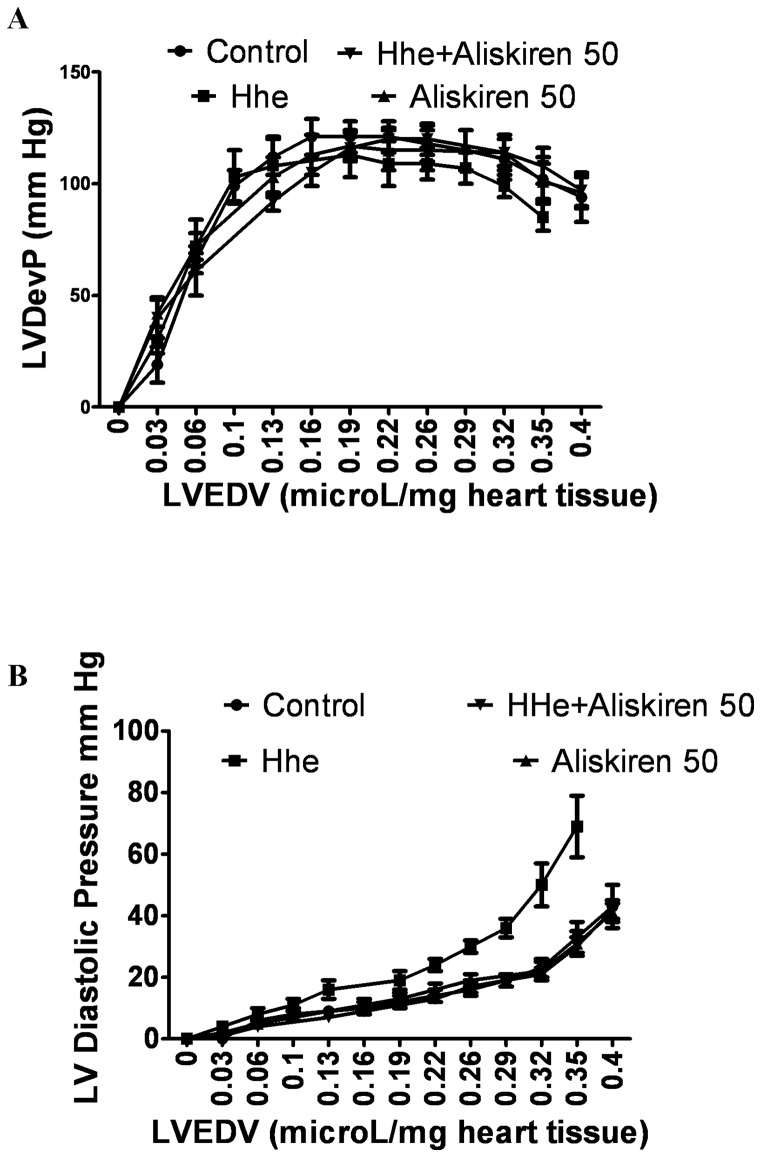
Effects of aliskiren on Hhe-induced diastolic dysfunction. (n = 5–7/group). A, the relation of balloon volume (normalized to heart weight) to left ventricular developed pressure. There were no differences between the groups. B shows the relation of balloon volume to left ventricular end diastolic pressure. There was a significant displacement of the pressure volume curve upwards in the Hhe group compared to all other groups (ANOVA; p<0.05). Hhe – Hhe-inducing diet; LVEDV – left ventricular end diastolic volume; and LVDevP – left ventricular developed pressure.

Figure 4 demonstrates the effects of aliskiren on cardiac systolic and diastolic function. Hhe worsened diastolic function as evidenced by an upward displacement of the diastolic pressure-volume relationship compared to control group, similar to our published studies in rats (ANOVA; p<0.05) [11]. Treatment with aliskiren in the dose of 50 mg/kg/day prevented diastolic dysfunction and normalized the pressure-volume relationship (ANOVA; p<0.05). There was no effect of Hhe or aliskiren on systolic function.

### Effects of aliskiren on matrix expression in cultured cardiac fibroblasts

**Figure 5:**
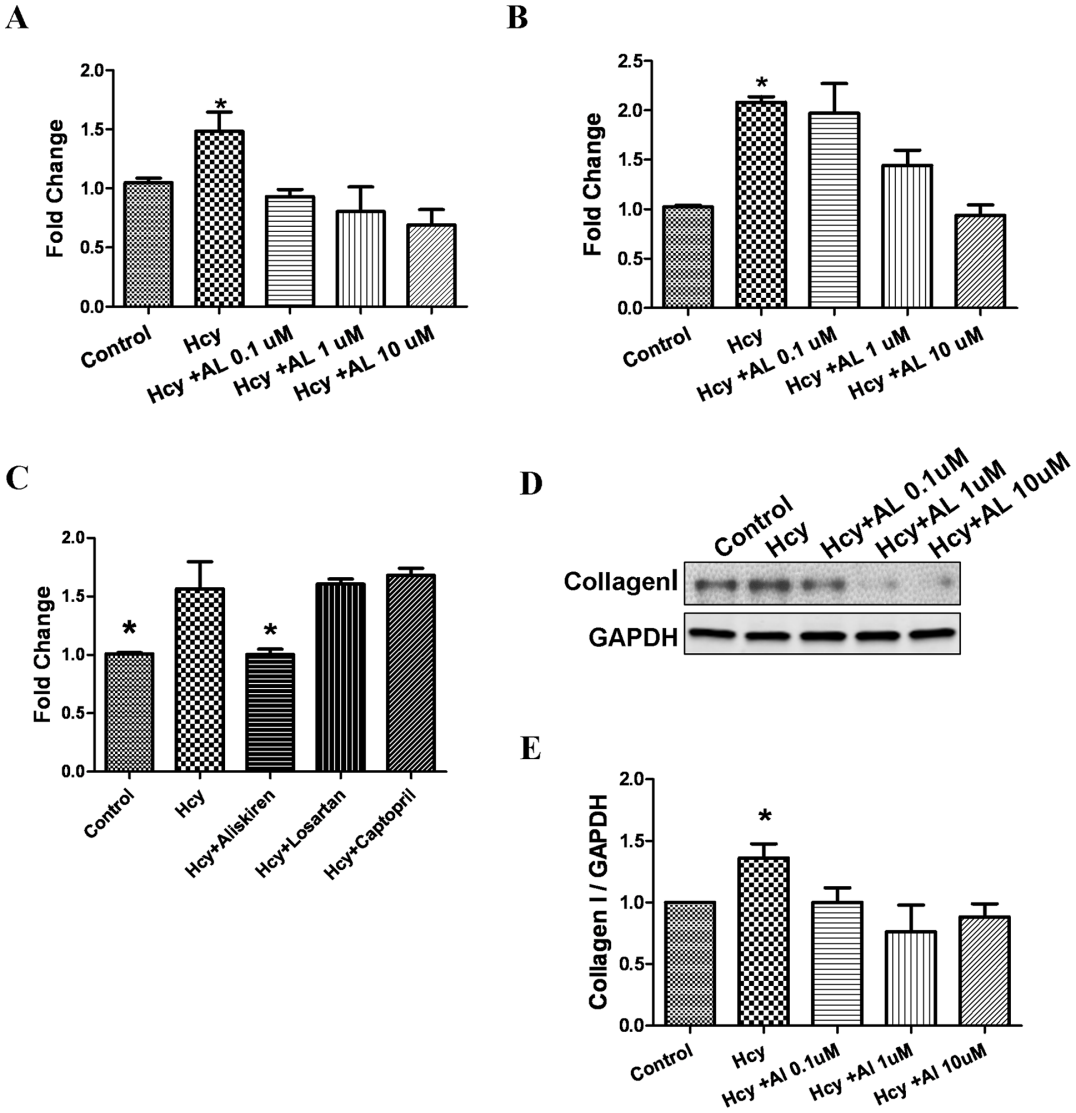
Effects of aliskiren on homocysteine-induced matrix expression in cultured cardiac fibroblasts. Fibroblasts were cultured in the presence or absence of D,L, homocysteine 200 µmol/L for 24 hours after pretreatment with varying concentrations of aliskiren (0.1–10 µmol/L). Real time PCR was done to measure relative expression of COLIA2 (alpha 2 chain of type I collagen; A) and COL3A1 (alpha 1 chain of type III collagen; B) genes. Figure 5C demonstrates the results of real time PCR to compare the effects of losartan and captopril with aliskiren on homocysteine induced expression of COL1A2 gene. Western blotting was conducted to measure the expression of collagen type I protein (D and E) Glyceraldehyde 3 phosphate dehydrogenase (GAPDH) was used as internal control. Hcy-D,L, homocysteine; AL-aliskiren; Los – losartan; and Cap – captopril. *p<0.05.

Figure 5 demonstrates the effects of aliskiren pretreatment on matrix gene expression in cardiac fibroblasts in the presence or absence of D, L, homocysteine. Homocysteine (400 µmol/L) increased mRNA levels for COL1A2 and COL3A1, which codes for the alpha 1 chain of collagen type III, the second most common collagen type observed in the heart (Figure 5A, 5B). Aliskiren reduced the expression of COL1A2 gene at low, intermediate and high doses, while the expression of COL3A1 was inhibited at intermediate and high doses. Losartan (0.1 mmol/L), an angiotensin II type I receptor blocker, and captopril (0.3 mmol/L), an angiotensin converting enzyme inhibitor, did not attenuate homocysteine-induced increase in COL1A2 gene expression (Figure 5C). The lower panels demonstrate the effect of homocysteine and aliskiren on collagen type I protein expression (Figure 5D and 5E). Homocysteine increased the expression of collagen type I protein in cardiac fibroblasts, while aliskiren attenuated the effects of homocysteine.

### Effects of Aliskiren on Signal Transduction in Cultured Cardiac Fibroblasts

**Figure 6:**
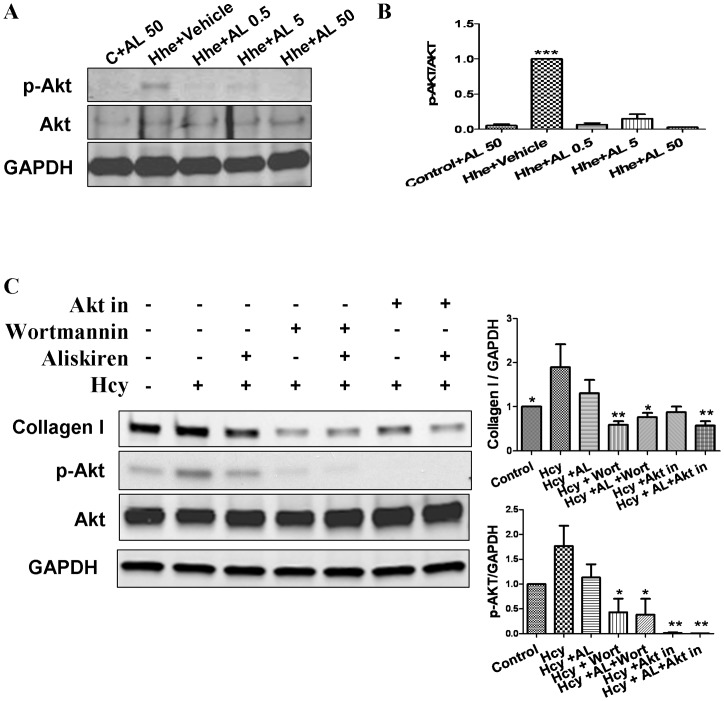
Role of Akt pathway in aliskiren's effects on attenuating collagen expression. A and B demonstrate the results of Western blotting for phospho- and total Akt in hearts of mice treated with various diets. C shows changes in the protein levels of collagen type I, phospho- and total Akt in response to 24 hours of exposure to D,L. Homocysteine (200 µmol/L) with or without pre-treatment with aliskiren 10 µmol/L (AL), wortmannin 1 µmol/L or Akt Inhibitor VIII 1 µmol/L. Hhe – Hhe-inducing diet; D,L, homocysteine (lower panel); AL 0.05– aliskiren 0.5 mg/kg/day; AL 5– aliskiren 5 mg/kg/day; AL 50– aliskiren 50 mg/kg/day; Wort – wortmannin; Akt in – Akt inhibitor VIII. *p<0.05; **p<0.01; and ***p<0.001.

As shown in Figure 6 (upper panel), phospho-Akt expression was significantly increased in heart tissues obtained from the Hhe group treated with vehicle compared to the control diet group, and was decreased to control values in the Hhe groups treated with aliskiren. The lower panel of Figure 6 demonstrates the effects of D, L Homocysteine (400 µmol/L) with and without addition of Aliskiren (1 µmol/L), wortmannin (1 µmol/L) and Akt Inhibitor VIII (1 µmol/L) on cultured cardiac fibroblasts. The increase in collagen type I protein expression induced by exposure to D, L, homocysteine was significantly decreased by aliskiren, wortmannin and Akt inhibitor. The combination of aliskiren with wortmannin or Akt inhibitor did not significantly increase the effect of either agent on homocysteine-induced collagen expression. The amount of phospho-Akt was significantly increased by homocysteine. Aliskiren, wortmannin, and the Akt Inhibitor VIII significantly decreased this effect. As in the case of collagen type I protein levels, the combination of aliskiren with the inhibitors of Akt pathway did not result in an additive effect on Akt phosphorylation.

## Discussion

Our results show that in a non-hypertensive, non-hypertrophic model of myocardial fibrosis and diastolic dysfunction [11], [16], long-term treatment with aliskiren significantly reduced matrix gene expression and improved diastolic function. Our *in vitro* results demonstrate that aliskiren directly attenuates the pro-fibrotic effects of homocysteine on cultured cardiac fibroblasts, and that the Akt pathway is involved in the beneficial effects of aliskiren. Cumulatively, our results suggest that direct renin inhibition with aliskiren exerts potent anti-fibrotic effects in the myocardium via both systemic effects on RAAS and direct effects on cardiac fibroblast biology, and thereby demonstrates the potential benefit of aliskiren in HFPEF.

Our results suggest that aliskiren can directly attenuate fibrotic mechanisms in the myocardium and normalize diastolic function in an established model of myocardial fibrosis. Although plasma angiotensin II levels and blood pressure were in the normal range in the Hhe group treated with vehicle, aliskiren dose dependently decreased both systolic and diastolic blood pressure and plasma angiotensin II levels on long-term treatment. A limitation of our *in vivo* study is that we did not directly compare the effect of aliskiren to an antihypertensive that acted independent of RAAS, or to another RAAS inhibitor. Since the blood pressure and angiotensin II levels in aliskiren groups were similar in both control and Hhe treated animals exposed to the highest does of aliskiren, it is not clear whether these systemic effects may have played a role in the anti-fibrotic effects of aliskiren. The observation that the heart weight to body weight ratios and cardiomyocyte size were similar in all groups suggests that the effects were independent of any load-induced ventricular hypertrophy. In addition, the anti-fibrotic effects of aliskiren were also observed at doses that did not alter blood pressure or angiotensin II levels. Recent investigations also suggest that the beneficial effects of aliskiren may be independent of blood pressure lowering. For example, in a mouse model of post-myocardial infarction heart failure, aliskiren was shown to have better effects on ventricular remodeling compared to a dose of hydralazine that produced comparable blood pressure lowering [17]. A similar study using high doses of aliskiren was shown to prevent the activation of extracellular signal regulated kinase and p38 mitogen activated protein kinase in the infarcted heart and to improve left ventricular function without decreasing the blood pressure [10].

Our results suggest that the myocardial matrix is a major target of aliskiren's cardiovascular effects. Myocardial type I collagen mRNA and protein levels induced by Hhe were reduced by exposure to lower doses of aliskiren. Our *in vitro* results corroborate our *in vivo* findings and suggest a direct anti-fibrotic effect of aliskiren on cardiac fibroblasts. Homocysteine-induced increase in the expression of collagen types I and III genes were suppressed by aliskiren in lower doses. Similar effects were observed on collagen type I protein levels secreted into the culture medium. Multiple studies have shown similar effects of renin, prorenin and their inhibition by inhibitors including aliskiren on matrix turnover in stromal cells. For example, He and coworkers have shown that (pro)renin receptor blockade resulted in anti-proliferative and anti-fibrotic effects in cultured rat mesangial cells [18]. Similarly, in rat and human mesangial cells, Huang and colleagues demonstrated a (pro)renin receptor mediated, angiotensin II-independent effect of renin in increasing transforming growth factor-β (TGF- β) and matrix gene expression [19]. In our study, losartan and captopril did not inhibit the effects of homocysteine on cardiac fibroblasts suggesting that the anti-fibrotic effect of aliskiren is independent of RAAS pathway. A recent study by Montes and colleagues in human fibroblasts isolated from normal and fibrotic lungs and demonstrated that renin, independent of angiotensin II, mediated TGF-β and collagen secretion. Our studies show that aliskiren has similar direct anti-fibrotic effects on cardiac fibroblasts.

Preclinical studies from our laboratories and those of others have shown that Hhe promotes myocardial fibrosis and dysfunction [12], [20], [21]. Epidemiological and clinical studies have also shown a link between Hhe and both diastolic and systolic heart failure [22], as well as between plasma homocysteine and plasma markers of matrix metabolism in subjects with normal and remodeled hearts [23]. We utilized this clinically relevant model to examine the effects of aliskiren on myocardial fibrosis. Multiple mechanisms such as oxidant stress, methylation, and direct incorporation of homocysteine into proteins or homocysteinylation, are proposed as pathogenic mechanisms of Hhe-induced cardiovascular dysfunction [24]. Interestingly, several studies have shown an interaction of the methionine-homocysteine cycle and RAAS. For example, *in vivo*, hyperhomocysteinemia has been demonstrated to enhance angiotensin II-induced vasoconstriction [25], and conversely, angiotensin II has been shown to exacerbate Hhe-induced changes in matrix accumulation and mechanics in small arteries [26]. Similarly, Hhe-induced changes in cerebrovascular and aortic reactivity are attenuated by ACEI [27]–[29]. The exact mechanism of this interaction is not known; a study by Suzuki and colleagues have shown that in cultured fibroblasts, homocysteine modulated the effects of angiotensin II on expression of the transcription factors GATA-4, nuclear factor of activated T cells and serum response factor [30]. Hence the myocardial effects of Hhe observed in our study may be dependent on homocysteine-induced activation of RAAS. The current study does not allow a clear separation of angiotensin II-mediated and angiotensin-II independent effects of aliskiren in myocardial fibrosis, since we did not examine the tissue RAAS in detail. However, since the effects of aliskiren were observed in cultured cardiac fibroblasts as well as with an *in vivo* dose which did not reduce plasma angiotensin II levels or blood pressure, it is likely that the effects of aliskiren were mediated by inhibition of (pro)renin receptor mediated and angiotensin II- independent effects of renin and prorenin. Multiple studies have demonstrated effects of prorenin and renin independent of the RAAS. In the initial reports by Nguyen on the (pro)renin receptor, it was demonstrated that renin-induced DNA synthesis was independent of enzymatic activity [31]. The (pro)renin receptor has been shown to modulate pro-fibrotic response in cultured mesangial cells [18] independent of angiotensin II [19]. Signal transduction via (pro)renin receptor is a major mode of cellular action of renin and prorenin independent of angiotensin II. *In vivo* studies on rats transgenic for the human (pro)renin receptor demonstrated that mitogen activated protein kinase activation and TGB-β expression was not attenuated by an ACEI, suggesting angiotensin II-independent MAPK activation modulated via the (pro)renin receptor [32].

Multiple signal transduction pathways including Phosphoinositide-3 kinase (PI3K)/Akt pathway has been shown to be induced by prorenin in cultured vascular smooth muscle cells [33]. Abekhoukh and colleagues demonstrated that in mice with hyperhomocysteinemia due to a genetically induced deficiency of the enzyme cystathionine beta synthase, a specific kinase termed dual-specificity tyrosine phosphorylation regulated kinase 1A modulated the PI3K/Akt pathway resulting in increased levels of phospho-Akt [34]. In another study, acute hyperhomocysteinemia was found to increase Akt phosphorylation in the rat hippocampus, while chronic hyperhomocysteinemia did not alter Akt phosphorylation [35]. In vitro studies have also demonstrated a direct effect of homocysteine on the PI3K/Akt pathway [36]. A study by Zou and colleagues demonstrated that homocysteine activated the Akt pathway and promoted proliferation of hepatic stellate cells [37]. Another study by Doronzo and colleagues demonstrated that in cultured vascular smooth muscle cells, homocysteine activated the PI3K/Akt pathways via the N-methyl-D-aspartate receptor, a glutamate-gated calcium ion channel; the increased in Akt phosphorylation was found to promote increased secretion of MMP-2. [38] Similarly, in murine macrophages, homocysteine increased Akt phosphorylation, and inhibition of Akt phosphorylation attenuated homocysteine induced MMP-9 expression [39], while in rat glomerular mesangial cells, homocysteine increased phosphorylation of PI3-kinase [40]. In these studies and others, homocysteine activated not only Akt, but also other signaling pathway such as ERK and p38MAPK. Hence hyperhomocysteinemia acts through the Akt signaling pathway as shown by our results; but the exact mechanisms of activation and cross-talk with other signaling pathways need further elucidation. Furthermore, the effect may be different in different cell types. Our study demonstrated that homocysteine activated the Akt pathway and that aliskiren's effects on preventing homocysteine induced matrix accumulation may be mediated via inhibiting activation of the Akt pathway. The effect of aliskiren on Akt activation is not well understood. Westermann and colleagues, in their study of aliskiren in a rodent model of myocardial infarction, demonstrated that myocardial phospho-Akt levels were increased after MI [10]. In this study, aliskiren decreased Akt phosphorylation and ameliorated cardiac remodeling, similar to the results observed in our study. The exact mechanisms by which renin inhibition would ameliorate signaling via Akt pathway have not been elucidated. Since renin can promote intracellular signaling by direct binding to the (pro)renin receptor independent of angiotensin receptor activation, it is possible that renin may have direct effect on kinases that promote signaling via the PI3K/Akt pathway.

## Conclusions

In conclusion, our results suggest that aliskiren exerts potent and direct anti-fibrotic effects in a clinically relevant model of myocardial fibrosis and diastolic dysfunction. Since current modes of RAAS antagonism may not provide a potent anti-fibrotic effect [7] and since ACEI and ARBs have not been shown to be of benefit in HFPEF, our results suggest that aliskiren as monotherapy may be useful in treating HFPEF.
